# Repair of calcified bicuspid aortic valves using living autologous aortic wall leaflets

**DOI:** 10.1016/j.xjtc.2024.03.011

**Published:** 2024-04-28

**Authors:** Timothy W. James, J. Hunter Mehaffey, Lawrence M. Wei, Rochus K. Voeller, Vinay Badhwar, J. Scott Rankin

**Affiliations:** a Department of Cardiac Surgery, St Joseph's Hospital, Tacoma, Wash; b Department of Cardiovascular and Thoracic Surgery, West Virginia University, Morgantown, WVa; c University of Minnesota, Minneapolis, Minn

**Figure:**
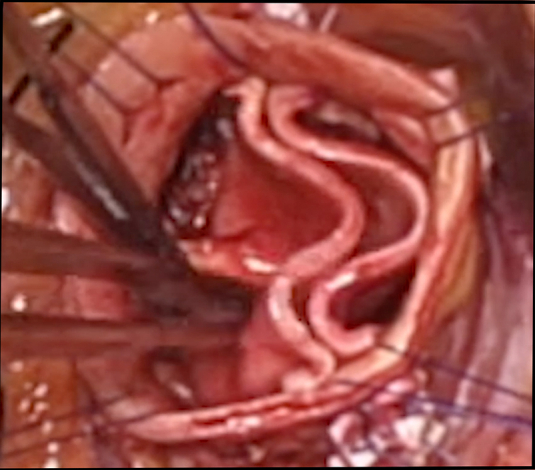
Autologous tissue valve for bicuspid valve repair created from aortic wall leaflets.

**Central Message**

A technique was developed to repair calcified bicuspid valves with a subannular annuloplasty ring and autologous aortic wall leaflets. Early clinical and echocardiographic results were excellent.

## Surgical Technique

Aortic valve repair for young patients with aortic insufficiency and reparable leaflets is now routine^1^^,^^2^ with demonstrable clinical benefits.^3^ Mild-to-moderate leaflet calcification can be managed with ultrasonic debridement (Video 1) or patches of autologous aortic wall (Video 2) with good intermediate-term results.^2^^,^^4^ However, more severe calcification is common in bicuspid aortic valve (BAV) disease, and often requires prosthetic valve replacement. A method of creating a living autologous tissue valve could be useful, and recent positive experiences with ascending aortic wall used as leaflet patches suggest that fully autologous valve reconstruction might be possible.^4^ This article describes techniques for managing calcification during BAV repair and documents the first human application of autologous aortic wall leaflets for complete autologous aortic valve reconstruction.

Based on computed tomography angiograms of normal aortic valves, aortic annuloplasty rings were developed,^5^ and as a byproduct, the geometric characteristics of aortic valve leaflets were defined. In unpublished porcine and human cadaver heart studies, leaflets of the proper shape and size were constructed from ascending aortic tissue, with free-edge length = valve diameter × 1.5, and geometric height = free-edge length × 0.75. With an internal annuloplasty ring positioned below the annulus as a geometric stent, the aortic wall leaflets were passed onto the annular sutures and secured above the valve annulus. After developing the method in porcine and human cadaver hearts, the technique was applied clinically.

Legal consultation determined that this new procedure primarily posed an issue of informed consent because no new devices were employed, and ample examples of leaflet patching already existed. After identifying an appropriate patient, detailed informed consent was provided about the new procedure, including theoretical advantages of autologous tissue, as well as limited follow-up data available. Consent also was obtained for publication of clinical data and video images. Approval of publication was obtained from our institutional review board (WVU #2005016064; approval date May 29, 2020; expiration date May 28, 2025). Videos in this article were recorded with 10-mm thoracoscopes and high-definition cameras, held in position by arms attached to the operating table. Videos, associated echocardiograms, and patient demographic data subsequently were de-identified and assembled with standard video production software.

**Figure 1:**
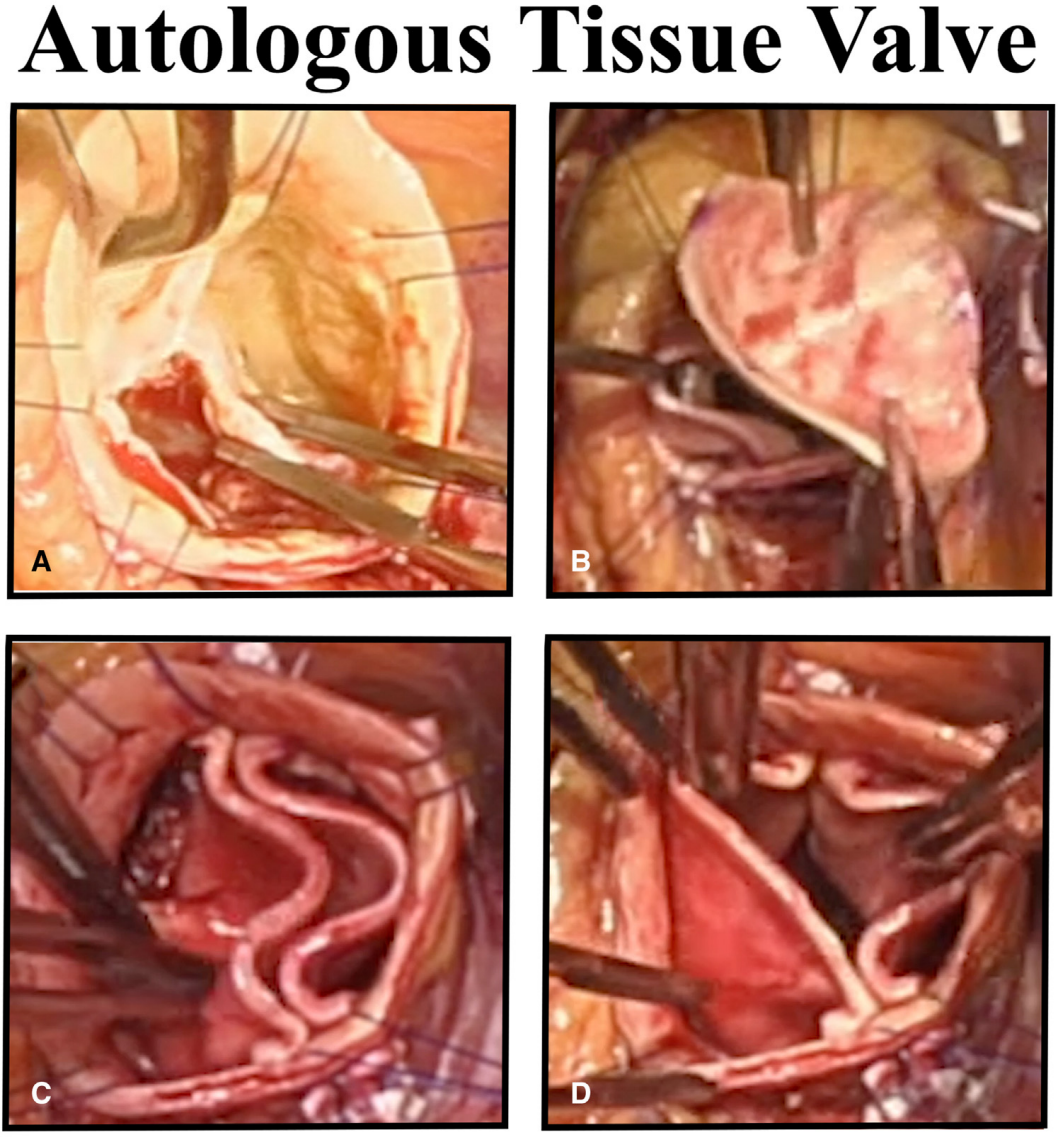
Steps in creating an autologous tissue valve. A, Calcified unicuspid valve is evident. B, Leaflet from ascending aortic tissue is being passed onto annular sutures. C, Autologous tissue valve is completed. D, Leaflets open well at the conclusion.

The patient undergoing complete autologous aortic valve leaflet reconstruction (Video 3) was a 51-year-old man with New York Heart Association functional class III heart failure from severe aortic stenosis and moderate insufficiency due to severely calcified unicuspid valve (Figure 1). His ascending aorta was 47 mm, and the valve gradient was 75 mm Hg peak and 42 mm Hg mean. Both the left-right and right noncommissures were fused (Video 3). The calcified leaflets were excised and debrided. The annulus was 27 mm, and a 25-mm bicuspid annuloplasty ring was selected for use as the valve stent. The ring established two 180° commissures with 2 equivalent circular annular segments. Both the anterior and posterior ring posts were sutured into the subcommissural triangles to the outside of the aorta. The ring was positioned below the annulus, and looping horizontal mattress sutures were placed around the body of the ring and up through the annulus without gaps.

**Figure 2:**
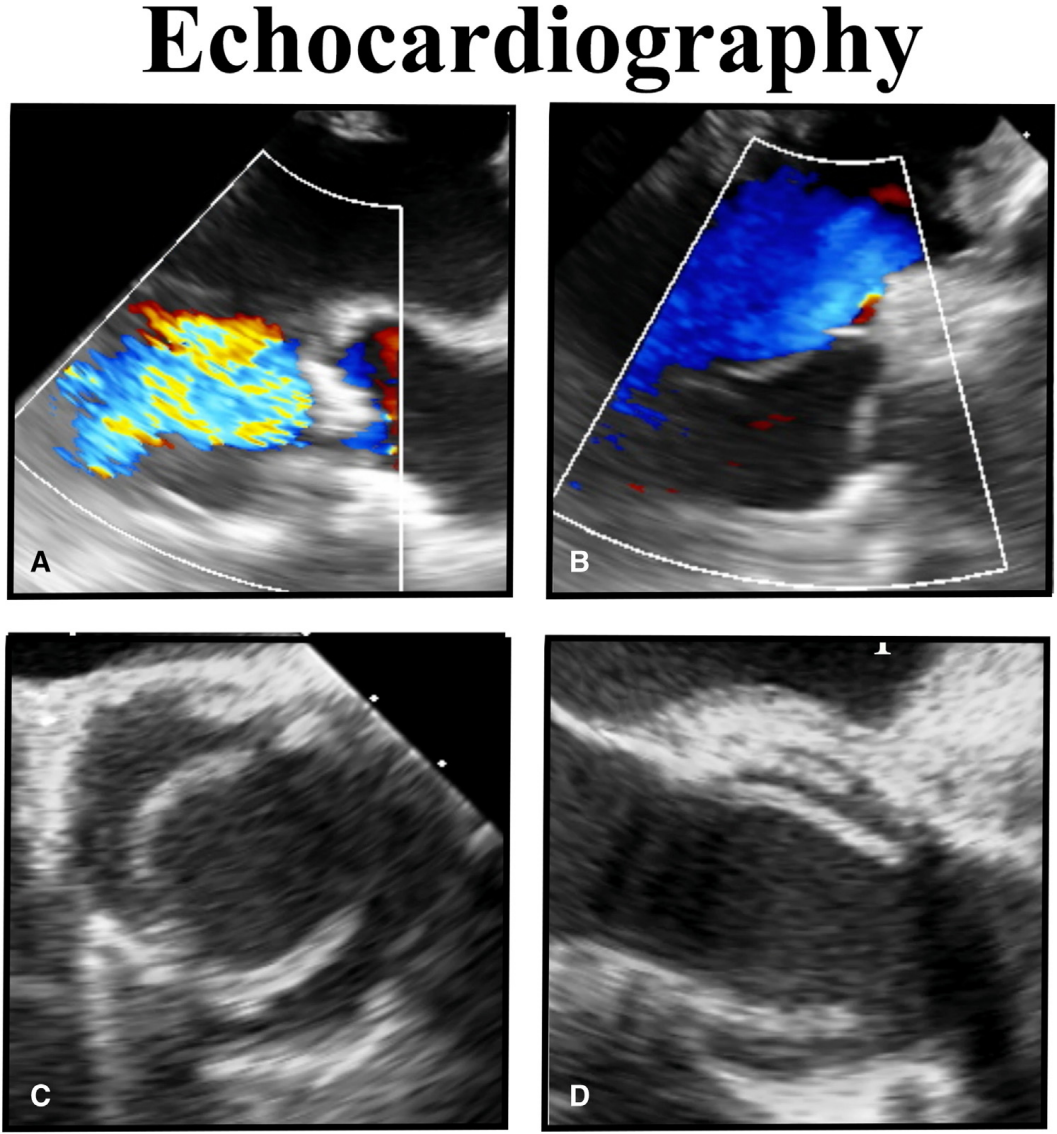
Echocardiography of autologous tissue valve. A, Calcified leaflets are moderately severely insufficient preoperatively. B, After repair, the autologous valve is fully competent. C, Repaired valve opens well during systole. D, Repaired aortic wall leaflets are tall and parallel during ejection.

The enlarged ascending aorta was excised, and 2 aortic leaflets were fashioned with free-edge lengths of half ring circumference, or 39 to 40 mm (Video 3). Geometric height was three-fourths of free-edge length or 30 mm. Both leaflets were passed down onto the annular mattress sutures, with the intima facing coaptation, and with fine polyethylene terephtalate pledgets above the leaflets. The external post sutures were secured, and all annular-leaflet looping sutures were tied. Relative to a 39-mm suture, both leaflet free-edge lengths looked good, with equal lengths and vertical heights. Both were trimmed to below the coronary ostia, and finally, commissural alignment sutures were placed. After repair, both leaflets moved well on echocardiograph (Figure 2), with good central flow, a 6 mm Hg mean gradient, excellent coaptation height, and no leak. At latest follow-up, valve function has remained excellent to beyond 8 months.

The association of calcified bicuspid or unicuspid valve with ascending aortic dilatation is common in younger patients. Modest calcification can be removed with ultrasonic debridement (Video 1), differentially applying energy proportional to tissue density, and follow-up of this approach has been excellent to 6 to 8 years.^2^ More serious localized leaflet calcification can be excised and repaired with an aortic wall patch (Video 2); this type of patch repair has been stable in more than 40 patients to 2 years.^4^ In extreme cases with heavy calcification, as in Video 3, complete leaflet reconstruction becomes necessary. Double-leaflet replacement has been employed previously, but with pericardium (https://www.ctsnet.org/article/autologous-pericardial-leaflet-replacement-bicuspid-aortic-valve-endocarditis) that routinely degenerated.

The ease and stability of autologous aortic leaflet placement in this patient suggest that the technique could be applied readily in appropriate candidates. Initially placing a bicuspid ring as a stent stabilizes the annulus and creates 180° annular geometry (independent of preoperative configuration), thus allowing use of 2 equal-sized aortic wall leaflets. The biologic argument for this approach is strong. Aortic wall is living autologous tissue that is accustomed to systemic pressure and gets its nutrition by luminal diffusion. Leaflet motion, transvalvular flow, and gradients seem excellent—with ample tissue available for leaflet substitution and minimal additional operative time. A larger series, longer-term follow-up, and careful echocardiographic assessment will be necessary for validation, but an autologous aortic tissue valve could contribute to future outcome improvement. Rather than answering “No” to complex BAV repair,^6^ going through the process of repair development^7^ could yield autologous techniques that might obviate future sacrifice of normal pulmonary valves.^8^

### Webcast

You can watch a Webcast of this AATS meeting presentation by going to: https://www.aats.org/resources/bicuspid-aortic-valve-replacem-7338.

**Figure:**
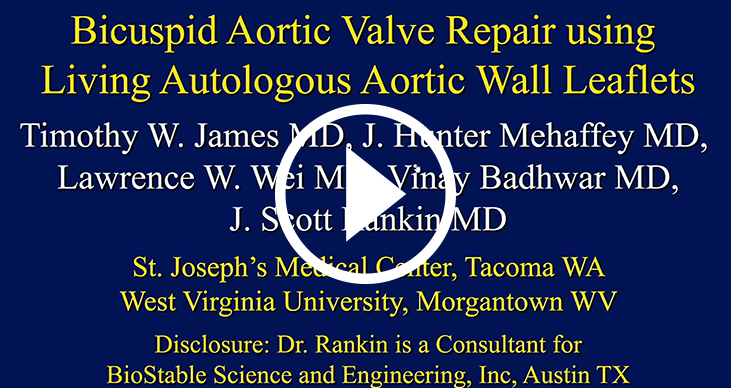

## Conflict of Interest Statement

Dr Rankin has been a consultant for BioStable Science and Engineering. All other authors reported no conflicts of interest.

The *Journal* policy requires editors and reviewers to disclose conflicts of interest and to decline handling manuscripts for which they may have a conflict of interest. The editors and reviewers of this article have no conflicts of interest.

## Supplementary Data

Video 1 Repair of a moderately calcified bicuspid aortic valve using geometric ring annuloplasty and an ultrasonic aspirator. Video available at: https://www.jtcvs.org/article/S2666-2507(24)00139-1/fulltext.

Video 2 Using a patch of aortic wall to replace a calcified raphe and cleft during unicuspid aortic valve repair. Video available at: https://www.jtcvs.org/article/S2666-2507(24)00139-1/fulltext.

Video 3 Bicuspid aortic valve repair using living autologous aortic wall leaflets. Video available at: https://www.jtcvs.org/article/S2666-2507(24)00139-1/fulltext.
