# Supplementary figures and images for: Repair of calcified bicuspid aortic valves using living autologous aortic wall leaflets
Source: JTCVS Tech. 2024 Apr 28;25:48–51. doi: 10.1016/j.xjtc.2024.03.011 (PMC11184624; doi:10.1016/j.xjtc.2024.03.011)

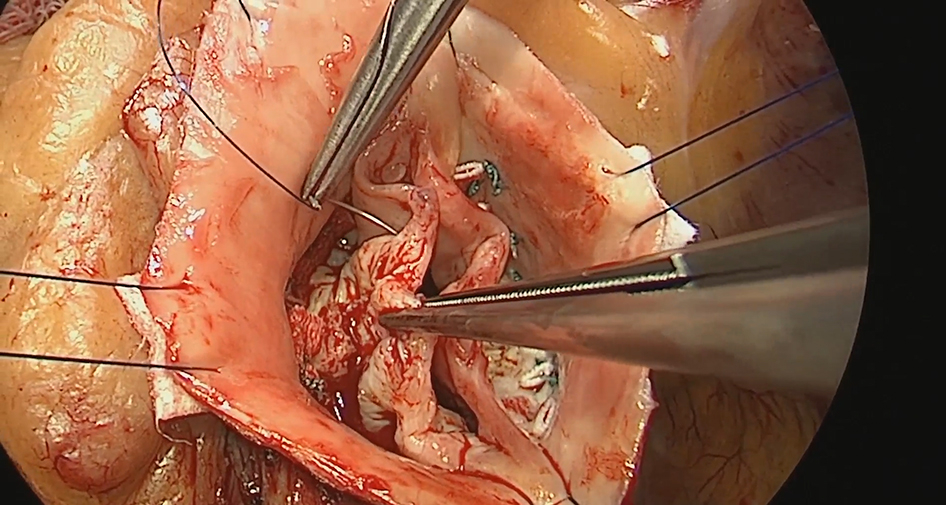

Supplement: Video 1 — Repair of a moderately calcified bicuspid aortic valve using geometric ring annuloplasty and an ultrasonic aspirator. Video available at: https://www.jtcvs.org/article/S2666-2507(24)00139-1/fulltext. [file fx2.jpg]

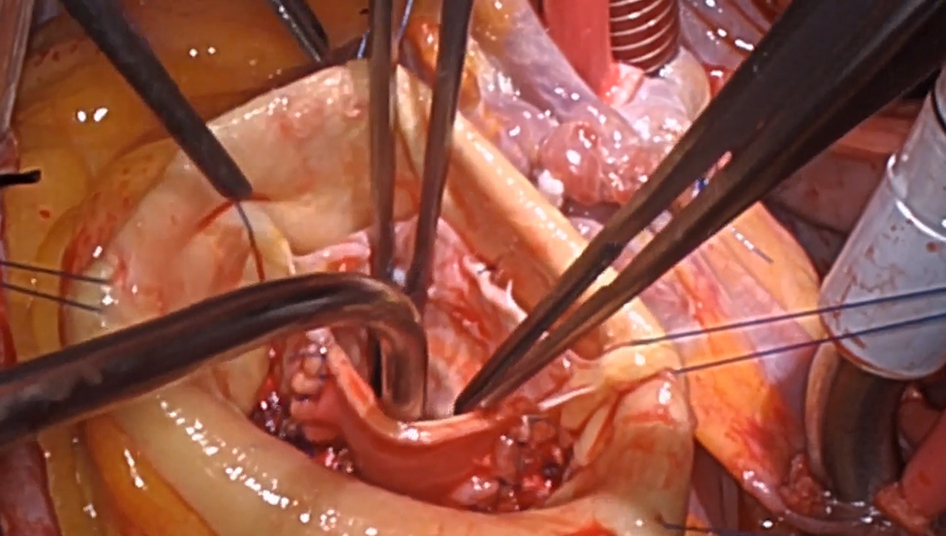

Supplement: Video 2 — Using a patch of aortic wall to replace a calcified raphe and cleft during unicuspid aortic valve repair. Video available at: https://www.jtcvs.org/article/S2666-2507(24)00139-1/fulltext. [file fx3.jpg]

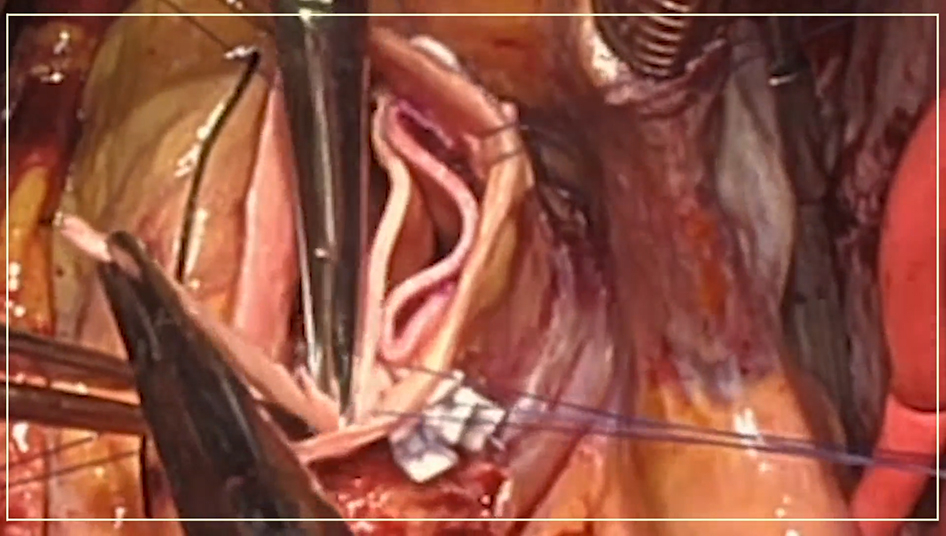

Supplement: Video 3 — Bicuspid aortic valve repair using living autologous aortic wall leaflets. Video available at: https://www.jtcvs.org/article/S2666-2507(24)00139-1/fulltext. [file fx4.jpg]
